# Visual outcomes and prognostic factors of vitrectomy for lamellar macular holes and epiretinal membrane foveoschisis

**DOI:** 10.1371/journal.pone.0247509

**Published:** 2021-02-22

**Authors:** Takashi Omoto, Yuichi Asahina, Han Peng Zhou, Ryosuke Fujino, Muneyuki Takao, Ryo Obata, Tatsuya Inoue, Ryo Asaoka, Maiko Maruyama-Inoue, Yasuo Yanagi, Kazuaki Kadonosono

**Affiliations:** 1 Department of Ophthalmology, The University of Tokyo, Tokyo, Japan; 2 Department of Ophthalmology and Micro-Technology, Yokohama City University, Kanagawa, Japan; Massachusetts Eye & Ear Infirmary, Harvard Medical School, UNITED STATES

## Abstract

**Purpose:**

To evaluate the visual outcomes of vitrectomy for lamellar macular hole (LMH) and epiretinal membrane (ERM) foveoschisis and to investigate the prognostic factor for postoperative visual acuity.

**Methods:**

We retrospectively reviewed 15 LMH and 17 ERM foveoschisis eyes that had undergone a standard three-port pars plana vitrectomy with (12 eyes) or without (20 eyes) cataract surgery. Best-corrected visual acuity (BCVA) at postoperative three months and the final visit were compared to the preoperative measurements. We investigated the relationship between BCVA at the final visit and baseline parameters (age, preoperative BCVA, the presence of epiretinal proliferation [EP] and ellipsoid zone [EZ] disruption). The best explanatory variables for the final BCVA were investigated using the corrected Akaike information criterion (AICc) model selection.

**Results:**

The mean age was 67.2 years. The mean follow-up duration was 30.7 months. Fifteen of 32 examined eyes were diagnosed as LMH and 17 eyes as ERM foveoschisis. Twelve eyes in LMH had EP and 13 eyes showed the disruption of EZ integrity. In total, BCVA significantly improved at 3 months postoperatively (p = 0.0013). A significant improvement was observed in ERM foveoschisis (p = 0.0085) but not in LMH group (p = 0.071). Comparing the BCVA between the baseline and the final visit, significant improvements were observed in total, ERM foveoschisis and LMH group (p<0.001, p<0.001 and p = 0.026, respectively). The optimal model for BCVA at the final visit included preoperative BCVA and the presence of EZ disruption (p<0.001 and p<0.001, respectively).

**Conclusion:**

Our results suggested that the final BCVA was dependent on preoperative BCVA and the presence of EZ disruption. Surgical indications might be warranted for LMHs with EZ disruption.

## Introduction

Recent advances in optical coherence tomography (OCT) in decades has provided us precise information of retinal imaging. These improvements have facilitated more detailed analyses, which have resulted in more accurate diagnoses of various vitreo-interface-associated disorders, including lamellar macular hole (LMH) [1].

LMH has been originally characterized by an irregular foveal contour, rupture of the inner foveal surface, dissociation between the inner and outer foveal retinas, and absence of a full-thickness retinal defect with relative preservation of the foveal photoreceptors [2]. Some investigators have focused attention on epiretinal proliferation (EP). EP is a non-tractional epiretinal membrane (ERM) with an unusual appearance compared to normal ERM [2]. EP was defined according to its characteristic properties, such as the absence of contractility, as suggested by histopathological analysis [3]. Recently, Govetto et al [4] suggested that the presence or absence of this peculiar EP alone was not sufficient to subclassify LMHs because EP is not specific to LMHs: eyes with ERMs and non-lamellar macular holes may also have this entity. Instead, they proposed that LMHs be divided into degenerative and tractional types according to morphological and functional characteristics. In general, EP and the discontinuous ellipsoid zone (EZ) were observed in degenerative LMH eyes. Recent studies reported poor surgical outcomes of LMHs with EP [5, 6] and other researches proposed the association between EZ disruption and poor visual outcomes [7, 8].

More recently, Hubschman et al. proposed new definitions of LMH to differentiate LMH from other similar conditions [9]. The proposed mandatory criteria of LMH were (1) irregular foveal contour; (2) foveal cavity with undetermined edges; (3) presence of at least one other sign evoking a loss of foveal tissue, pseudo-operculum, thinning of the fovea at the centre or around. It was also proposed that “ERM foveoschisis” and “macular pseudohole (MPH)” were defined as similar conditions to LMH. ERM foveoschisis definition is based on two mandatory criteria; (1) presence of ERM; (2) presence of schisis at the Henle’s fiber layer (HFL). Mandatory criteria of MPH were: (1) foveal centre sparing ERM; (2) retinal thickening; (3) verticalized or steepened foveal profile. In addition, they proposed to use the term “EP” instead of “lamellar hole-associated EP”. Indeed, uniform definition and terminology enable to precisely research LMH and other similar clinical entities (ERM foveoschisis and MPH), however there was no report to investigate surgical outcomes according to these newly defined entities.

The purpose of the current study was to evaluate the visual outcomes of pars plana vitrectomy for LMH and ERM foveoschisis at 3 months and at the final visit after the surgery. Furthermore, we divided patients into subgroups depending on (1) if they were “LMH or ERM foveoschisis”, (2) if they were “with or without EP” and (3) if they were “with or without the disruption of EZ”. The surgical outcomes of the subgroups were also compared.

## Methods

This study was approved by the Research Ethics Committee of the Graduate School of Medicine and Faculty of Medicine at The University of Tokyo. Written consent was provided by patients for their information to be stored in the hospital database and used for research. This study was performed according to the tenets of the Declaration of Helsinki.

In this observational case study, we retrospectively reviewed the medical records of patients with LMH and ERM foveoschisis. Patients with a history of advanced glaucoma or retinal disease such as diabetic retinopathy, retinal vein occlusion, or macular degeneration were excluded from the current study.

**Fig 1:**
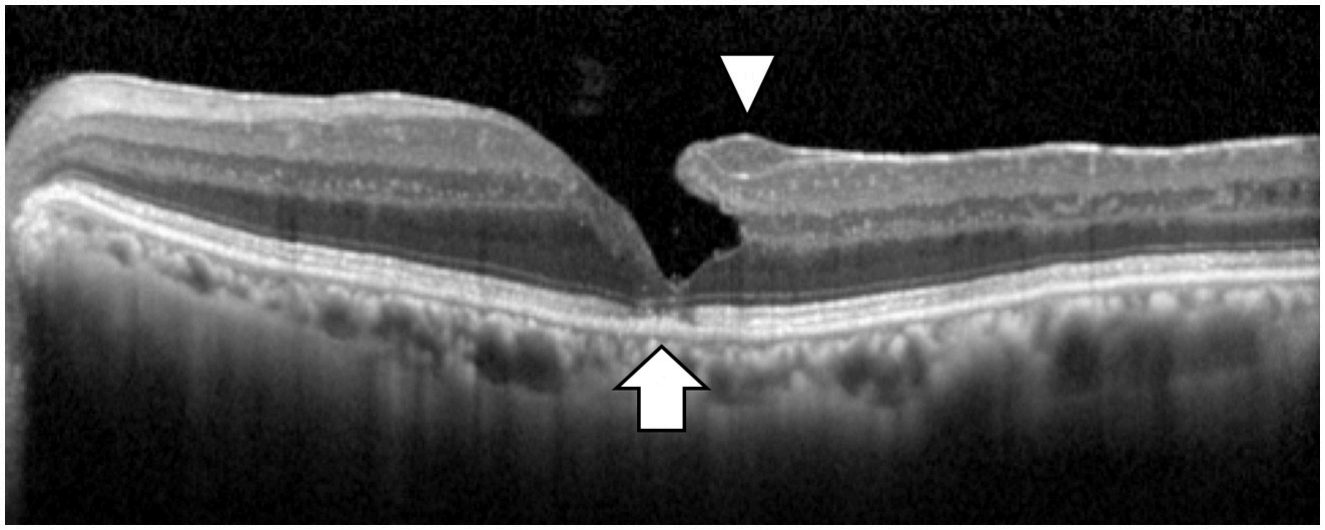
A representative OCT image of EP and EZ disruption in an eye with LMH. The arrow head shows EP and the arrow shows EZ disruption. EP: epiretinal proliferation, EZ: ellipsoid zone, LMH: lamellar macular hole.

All patients underwent Spectralis OCT (Heidelberg Engineering, Heidelberg, Germany) examination. All OCT images consisted of line scans (horizontal and vertical) with the scan size of 30 degrees. The specific morphological features used to classify LMH included (1) irregular foveal contour; (2) foveal cavity with undetermined edges; (3) presence of at least one other sign evoking a loss of foveal tissue, pseudo-operculum, thinning of the fovea at the centre or around. On the other hand, ERM foveoschisis was identified based on the presence of ERM and presence of schisis at the Henle’s fiber layer (HFL) (between the outer plexiform and outer nuclear layers) [4]. The presence of EP and the disruption of EZ integrity were also assessed in all examined eyes (Fig 1). All OCT images were carefully analyzed by two independent examiners (YA, RF). The results were then verified by another examiner. If the second examiner did not agree with the first examiner, a panel discussion was held until a final diagnosis was achieved.

All patients underwent a standard three-port pars plana vitrectomy (25-gauge). In all cases, the ILM was peeled inside the arcade area as large as possible using diluted indocyanine green dye to enhance visualization during ILM peeling. EP was completely removed in eyes with EP. Gas tamponade was performed at the discretion of the surgeons with air or sulfur-hexafluoride (SF6) gas.

Best corrected visual acuity (BCVA) were used as the logarithm of the minimum angle of resolution manner for statistical analysis. The paired Wilcoxon signed rank test was used to compare preoperative and postoperative BCVA (at 3months and at final visit after surgery) in total eyes and each subgroup. Moreover, we investigated the relationship between BCVA at the final visit and baseline parameters (age, preoperative BCVA, the presence of EP and EZ disruption). Using the corrected Akaike information criterion (AICc) model selection, we investigated which baseline parameters were the best explanatory variables for BCVA at the final visit.

All statistical analyses were performed using the statistical programming language R (R version 3.4.3; R foundation for Statistical Computing, Vienna, Austria).

## Results

In the current study, 32 eyes of 32 patients (12 males and 20 females) were enrolled. The mean age of the patients was 67.2 ± 11.0 years. The mean follow-up duration was 30.7 ± 18.7 (ranged from 12 to 72) months. All the examined eyes were followed at least 12 months after vitrectomy surgery. 12 eyes underwent vitrectomy combined with phacoemulsification cataract surgery. The remaining 20 eyes underwent vitrectomy alone and subsequent cataract surgery between the postoperative 3 month and the final visit for the phakic eyes. Patient characteristics are summarized in Table 1.

**Table 1:** The demographic data of the patients.

	Total	LMH	ERM foveoschisis	P values
Number of eyes	32	15	17	
Age (years old)	67.2 ± 11	71.3 ± 6.7	63.6 ± 12.9	0.064
Sex (male)	12	6	6	1.0
Combined vitrectomy and cataract surgery	12	6	6	1.0
Follow-up duration (month)	30.7 ± 18.7	32.4 ± 21.7	29.1 ± 16	0.99
EZ disruption (+)	13	13	0	<0.001*
EP (+)	12	12	0	<0.001*
The preoperative refractive error (D)	-3.4 ± 5.6	-3.4 ± 6.0	-3.4 ± 5.3	0.99
Highly myopic eyes (<-5 diopter)	10	4	6	0.71
The preoperative BCVA (LogMAR)	0.25 ± 0.23	0.31 ± 0.22	0.2 ± 0.23	0.064

Values were shown as mean ± standard deviation.

P values were calculated using Wilcoxon rank sum test and Fisher’s exact test, appropriately.

*, shows the statically significant difference between the subgroups.

EZ: ellipsoid zone, EP: epiretinal proliferation.

Fifteen of 32 eyes were diagnosed as LMH and 17 eyes as ERM foveoschisis. Twelve eyes in LMH eyes had EP and 13 eyes showed the disruption of EZ. The mean preoperative BCVA in total 32 eyes was 0.25 ± 0.23. Mean preoperative BCVA in LMH eyes was 0.31 ± 0.22 and that in ERM foveoschisis was 0.20 ± 0.23, suggesting no significant difference between the groups (p = 0.13, Wilcoxon rank sum test). There was no significant difference in the preoperative BCVA between the presence and absence of EP (p = 0.60, Wilcoxon rank sum test). Furthermore, no significant difference was shown in the preoperative BCVA between the presence and absence of EZ disruption (p = 0.084).

**Fig 2:**
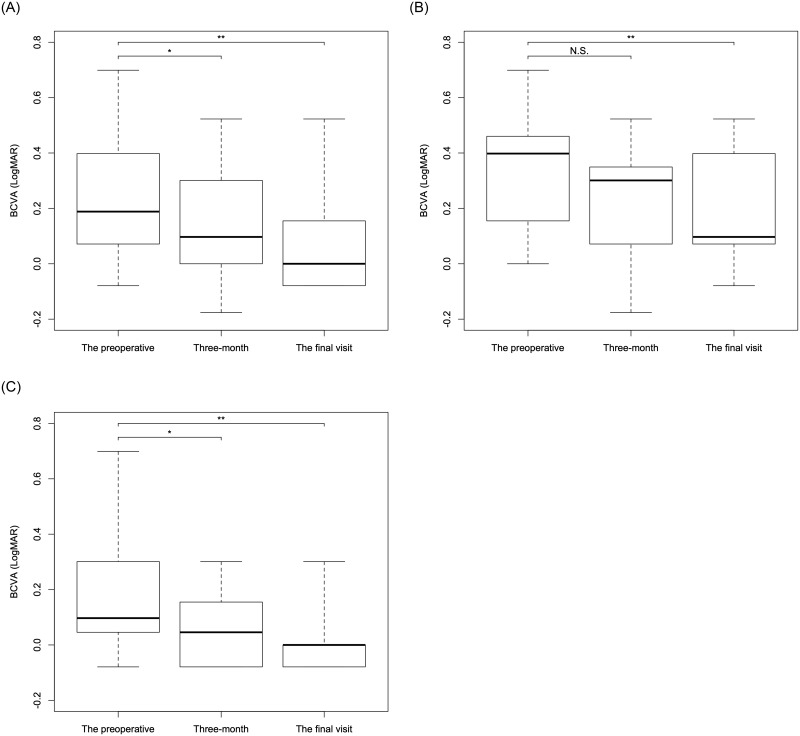
The changes of visual acuity over time in total and in each subgroup. The boxplots of visual acuity at each time point were shown in total (A) and in LMH (B) and ERM foveoschisis (C) subtypes. BCVA significantly improved at 3 months and at the final visit after the surgery in total (p = 0.0013 and p<0.001: A). There was no significant improvement at 3 months after the surgery in LMH (p = 0.071: B) however significant VA improvement was observed in ERM foveoschisis (p = 0.0085: C). Significant improvements were observed at final visit in both LMH and ERM foveoschisis (p = 0.026 and p<0.001, respectively, B and C). *, shows the significant difference between the preoperative and 3-month values. **, shows the significant difference between the preoperative and the final visit values. BCVA: best corrected visual acuity, N.S.: not significant.

BCVA significantly improved at 3 months (0.14 ± 0.20) and at the final visit (0.088 ± 0.19) after vitrectomy surgery overall (p = 0.0013, p<0.001, paired Wilcoxon signed rank test: Fig 2A). There was no significant improvement at 3 months after surgery in LMH (p = 0.071: Fig 2B) however significant VA improvement was observed in ERM foveoschisis (p = 0.0085: Fig 2C). Significant improvements were observed at final visit in both groups (p = 0.026 and p<0.001, respectively, paired Wilcoxon signed rank test: Fig 2B and 2C), although all the cases had undergone cataract extraction by the time.

**Fig 3:**
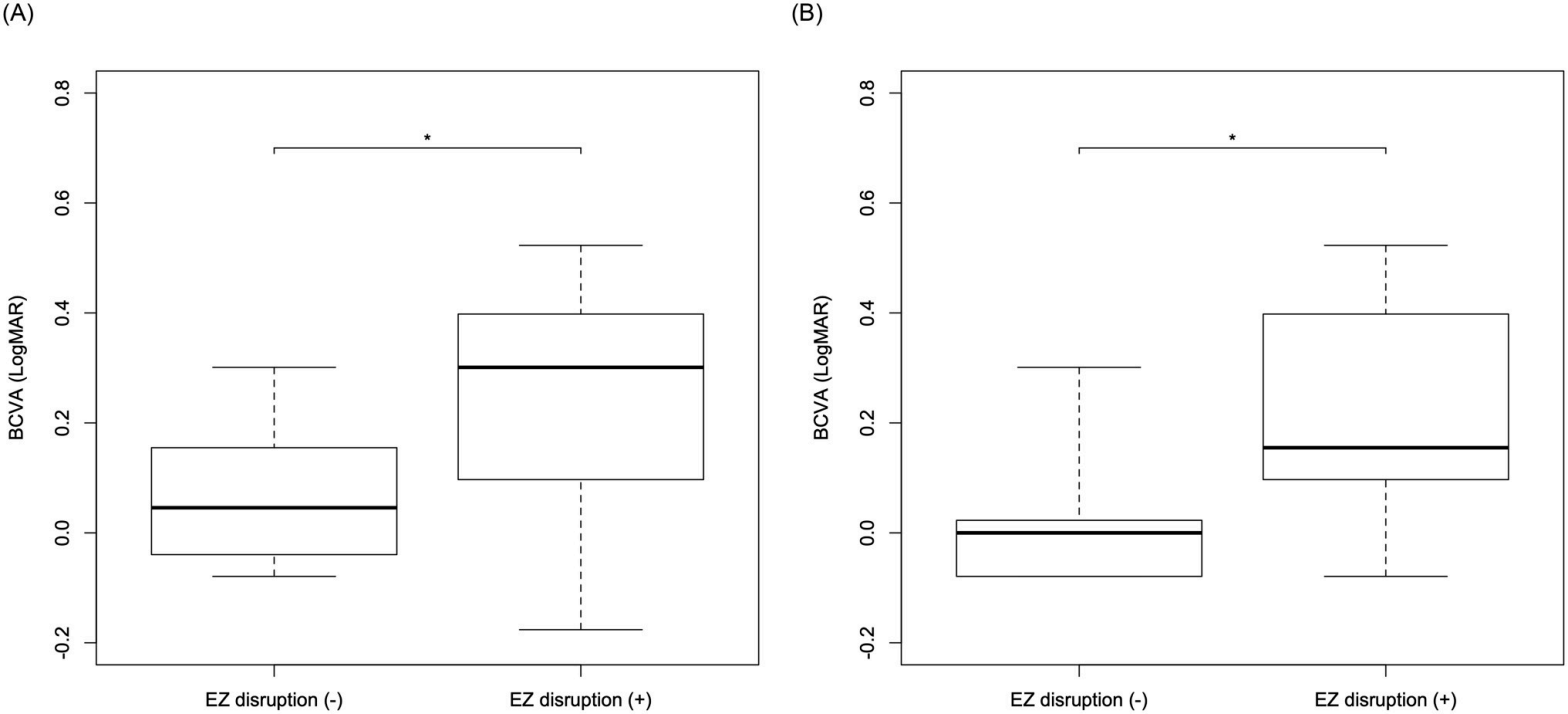
The three-month and the final BCVA in patients with or without EZ disruption. There was a significant difference in the three-month (A) and the final (B) BCVA between patients with or without EZ disruption (*p* = 0.017 and p<0.001, respectively, linear regression analysis). BCVA: best corrected visual acuity, LMH: lamellar macular hole, EZ: ellipsoid zone.

Table 2 shows the correlation between BCVA at the final visit and baseline parameters. The preoperative BCVA and the presence of EZ disruption were significantly related to the BCVA at the final visit (p<0.001, respectively). Furthermore, as the result of AICc model selection, the optimal model for the postoperative BCVA at the final visit included the preoperative BCVA and the presence of EZ disruption (AICc = -32.2). Worse preoperative BCVA and the presence of EZ disruption were correlated with worse postoperative BCVA at the final visit (Table 2). In fact, significant differences between EZ+ and EZ- groups were shown both in postoperative BCVA at 3 months (p = 0.017: Fig 3A) and at the final visit (p<0.001: Fig 3B). On the other hand, no significant differences between EP+ and EP- groups were observed both in postoperative BCVA at 3 months and at the final visit (p = 0.12, p = 0.063, linear regression analysis).

**Table 2:** Relationship between baseline parameters and the final BCVA.

	Univariate analysis			The optimal model		
Variables	Coefficient	SE	P value	Coefficient	SE	P value
Age	0.0051	0.0030	0.096	N.S.	N.S.	N.S.
The preoperative BCVA (LogMAR)	0.49	0.12	<0.001*	0.39	0.11	0.0011*
The presence of EP	0.13	0.065	0.063	N.S.	N.S.	N.S.
The presence of EZ disruption	0.21	0.057	<0.001*	0.16	0.050	<0.001*

Values were calculated using multivariate linear regression model.

The variables for the optimal model were determined using Akaike information criterion model selection.

*, shows p value < 0.05.

BCVA: best corrected visual acuity, SE: standard error, EP: epiretinal proliferation, EZ: ellipsoid zone, N.S.: not selected.

## Discussion

Previous studies have reported conflicting visual and anatomical results following LMH surgery, with fairly poor visual outcomes [10–14]. Since the follow-up periods in these studies ranged from six months to over two years, it was possible that the variability of follow-up period influenced on the conflicting results. In the current study, in total, BCVA improved from preoperative 0.23 to 0.14 at 3 months and 0.088 at the final visit with mean follow-up duration of 30.7 ± 18.7 months (at least 12 months), which is relatively long-term follow-up compared to previous researches. In subgroup analysis, BCVA improved from preoperative 0.20 to 0.063 at 3 months and -0.0047 at the final visit in ERM foveoschisis group while from preoperative 0.31 to 0.23 at 3 months and 0.19 at the final visit in LMH group. A recent report by Choi WS et al. showed a significant improvement from 0.48 to 0.16 (14.9 months) in eyes without EP and no significant improvement from 0.50 to 0.40 (23.4 months) in eyes with EP [15]. Another recent report by Figueroa showed that BCVA significantly improved from 0.38 to 0.18 in tractional type LMH and from 0.56 to 0.39 in degenerative type LMH [8]. Despite the difference of the baseline values, the improvement of BCVA in our study was comparable to these previous reports.

In our subgroup analysis, significant visual improvements were observed in both ERM foveoschisis and LMH groups at the final visit, while there was not at 3 months after the surgery in LMH group. It appears that the BCVA improvement occurred in ERM foveoschisis group earlier than LMH group. Figueroa et al. recently reported functional and anatomical outcomes after vitrectomy surgery in LMHs [8]. It was suggested that better postoperative BCVA was observed in tractional type, while the anatomical healing was delayed in this type, supporting the idea that LMH and ERM foveoschisis are considered as two distinct clinical entities [4, 16]. However, the present study enrolled the eyes that had undergone only PPV therefore future study is needed to clarify the functional and anatomical outcomes in eyes with LMH and ERM foveoschisis.

Lee CS et al. reported that a disrupted IS / OS junction (EZ disruption) was one of predictors of surgical outcome using multivariate linear regression for 30 LMH eyes [7]. Figueroa et al. also indicated that worse preoperative VA and outer retinal disruption (discontinuous EZ or external limiting membrane) were robust indicators of worse postoperative VA in eyes with LMH [8]. Interestingly, in their study, the majority of eyes in tractional LMH recovered foveal microstructure almost completely, without evidence of disruption in retinal layers and/or apparent tissue loss [8]. However, in our study, all cases with EZ disruption were in LMH group (Table 1), which might be contributed to the photoreceptor compromise. Consistent with these previous researches, our current result also suggested EZ disruption was closely related to postoperative VA (p<0.001: Table 2).

EP was found in 39.4% of all examined eyes, and was found in 81.3% of eyes in LMH. We found no significant differences in the preoperative BCVA and at 3 months and at the final visit between EP+ and EP- groups. Inconsistent with our result, some previous studies suggested significant visual improvements in eyes without EP, compared with eyes with EP in LMH eyes [14, 15]. This discrepancy might possibly be due to the difference of follow-up period or sample size of the studies. A recent meta-analysis by Xu et al. also confirmed that patients without EP had better postoperative visual acuity than patients with EP [6]. In the cases with EP, good surgical outcomes of sparing the ILM peeling of fovea were reported in some studies [17, 18]. However, our current results suggested preoperative BCVA and the presence of EZ disruption were significantly correlated to postoperative VA at final visit but the presence of EP was not.

Among the studies that reported a significant difference in visual outcomes between patients with EP+ and EP-, Choi WS et al. [15] recommended vitrectomy for patients who had progressive, disabling visual loss and an increase in EZ disruption, which is often seen in eyes with EP patients. Although our results are different from previous studies in that the presence of EP was not significantly correlated to postoperative VA, we agree that vitrectomy should be recommended for those with progressive visual loss, especially considering that preoperative VA was closely correlated to postoperative VA.

Limitations of our study include its retrospective nature; therefore, the details of the surgical procedures and follow-up protocols were not standardized. Takahashi et al. recently reported the surgical outcomes of EP embedding technique [19]. As a result, good anatomical and functional outcomes were observed, suggesting the effectiveness of this technique to treat LMHs. The optimal treatment for each type (LMH, ERM foveoschisis and MPH) should be established in the future studies. Moreover, a larger cohort size must be evaluated to obtain more accurate comparisons.

In conclusion, among the baseline parameters, the preoperative BCVA and the disruption of EZ were significantly correlated to postoperative BCVA, whereas the presence of EP was not. Although further studies are required to determine surgical indications, it is suggested that poor visual outcome might be simply dependent on the presence of EZ disruption at baseline in eyes with LMH.

## References

[1] Mirza RG, Johnson MW, Jampol LM. Optical coherence tomography use in evaluation of the vitreoretinal interface: a review. Surv Ophthalmol. 2007;52(4):397–421. 10.1016/j.survophthal.2007.04.007 17574065

[2] Witkin AJ, Ko TH, Fujimoto JG, Schuman JS, Baumal CR, Rogers AH, et al Redefining lamellar holes and the vitreomacular interface: an ultrahigh-resolution optical coherence tomography study. Ophthalmology. 2006;113(3):388–97. 10.1016/j.ophtha.2005.10.047 16513456 PMC1940046

[3] Compera D, Entchev E, Haritoglou C, Scheler R, Mayer WJ, Wolf A, et al Lamellar Hole-Associated Epiretinal Proliferation in Comparison to Epiretinal Membranes of Macular Pseudoholes. Am J Ophthalmol. 2015;160(2):373–84.e1. 10.1016/j.ajo.2015.05.010 25982970

[4] Govetto A, Dacquay Y, Farajzadeh M, Platner E, Hirabayashi K, Hosseini H, et al Lamellar Macular Hole: Two Distinct Clinical Entities? Am J Ophthalmol. 2016;164:99–109. 10.1016/j.ajo.2016.02.008 26898164

[5] Ko J, Kim GA, Lee SC, Lee J, Koh HJ, Kim SS, et al Surgical outcomes of lamellar macular holes with and without lamellar hole-associated epiretinal proliferation. Acta Ophthalmol. 2017;95(3):e221–e6. 10.1111/aos.13245 27647708

[6] Xu H, Qin L, Zhang Y, Xiao Y, Zhang M. Surgery outcomes of lamellar macular eyes with or without lamellar hole-associated epiretinal proliferation: a meta-analysis. BMC Ophthalmol. 2020;20(1):345 10.1186/s12886-020-01617-4 32842986 PMC7448992

[7] Lee CS, Koh HJ, Lim HT, Lee KS, Lee SC. Prognostic factors in vitrectomy for lamellar macular hole assessed by spectral-domain optical coherence tomography. Acta Ophthalmol. 2012;90(8):e597–602. 10.1111/j.1755-3768.2012.02456.x 22632460

[8] Figueroa MS, Govetto A, Steel DH, Sebag J, Virgili G, Hubschman JP. PARS PLANA VITRECTOMY FOR THE TREATMENT OF TRACTIONAL AND DEGENERATIVE LAMELLAR MACULAR HOLES: Functional and Anatomical Results. Retina. 2019;39(11):2090–8. 10.1097/IAE.0000000000002326 30312255

[9] Hubschman JP, Govetto A, Spaide RF, Schumann R, Steel D, Figueroa MS, et al Optical coherence tomography-based consensus definition for lamellar macular hole. Br J Ophthalmol. 2020;104(12):1741–7. 10.1136/bjophthalmol-2019-315432 32107208

[10] Garretson BR, Pollack JS, Ruby AJ, Drenser KA, Williams GA, Sarrafizadeh R. Vitrectomy for a symptomatic lamellar macular hole. Ophthalmology. 2008;115(5):884–6 e1. 10.1016/j.ophtha.2007.06.029 18067968

[11] Androudi S, Stangos A, Brazitikos PD. Lamellar macular holes: tomographic features and surgical outcome. Am J Ophthalmol. 2009;148(3):420–6. 10.1016/j.ajo.2009.04.009 19493523

[12] Witkin AJ, Castro LC, Reichel E, Rogers AH, Baumal CR, Duker JS. Anatomic and Visual Outcomes of Vitrectomy for Lamellar Macular Holes. Investigative Ophthalmology & Visual Science. 2009;50(13):6052-. 10.3928/15428877-20100426-04 20438044

[13] Casparis H, Bovey EH. Surgical treatment of lamellar macular hole associated with epimacular membrane. Retina. 2011;31(9):1783–90. 10.1097/IAE.0b013e31820a6818 21540765

[14] Figueroa MS, Noval S, Contreras I. Macular structure on optical coherence tomography after lamellar macular hole surgery and its correlation with visual outcome. Can J Ophthalmol. 2011;46(6):491–7. 10.1016/j.jcjo.2011.09.011 22153635

[15] Choi WS, Merlau DJ, Chang S. Vitrectomy for Macular Disorders Associated with Lamellar Macular Hole Epiretinal Proliferation. Retina. 2018;38(4):664–9. 10.1097/IAE.0000000000001591 28301339

[16] Gaudric A, Aloulou Y, Tadayoni R, Massin P. Macular pseudoholes with lamellar cleavage of their edge remain pseudoholes. Am J Ophthalmol. 2013;155(4):733–42, 42.e1–4. 10.1016/j.ajo.2012.10.021 23312734

[17] Ho TC, Ho AY, Chen MS. Reconstructing Foveola by Foveolar Internal Limiting Membrane Non-Peeling and Tissue Repositioning for Lamellar Hole-Related Epiretinal Proliferation. Sci Rep. 2019;9(1):16030 10.1038/s41598-019-52447-4 31690760 PMC6831694

[18] Morescalchi F, Russo A, Gambicorti E, Cancarini A, Scaroni N, Bahja H, et al Peeling of the Internal Limiting Membrane with Foveal Sparing for Treatment of Degenerative Lamellar Macular Hole. Retina. 2020;40(6):1087–93. 10.1097/IAE.0000000000002559 31107710

[19] Takahashi K, Morizane Y, Kimura S, Shiode Y, Doi S, Okanouchi T, et al Results of lamellar macular hole-associated epiretinal proliferation embedding technique for the treatment of degenerative lamellar macular hole. Graefe’s Archive for Clinical and Experimental Ophthalmology. 2019;257(10):2147–54. 10.1007/s00417-019-04425-9 31342148

